# Recommendations for the optimal use of bone forming agents in osteoporosis

**DOI:** 10.1007/s40520-024-02826-3

**Published:** 2024-08-09

**Authors:** Nicola Veronese, Karine Briot, Nuria Guañabens, Ben Hur Albergaria, Majed Alokail, Nasser Al-Daghri, Angie Botto-van Bemden, Olivier Bruyère, Nansa Burlet, Cyrus Cooper, Elizabeth M. Curtis, Peter R. Ebeling, Philippe Halbout, Eric Hesse, Mickaël Hiligsmann, Bruno Muzzi Camargos, Nicholas C. Harvey, Adolfo Diez Perez, Régis Pierre Radermecker, Jean-Yves Reginster, René Rizzoli, Heide Siggelkow, Bernard Cortet, Maria Luisa Brandi

**Affiliations:** 1 Geriatric Unit, Department of Internal Medicine and Geriatrics, University of Palermo, Via del Vespro 141, 90127 Palermo, Italy; 2 Chair for Biomarkers of Chronic Diseases, Biochemistry Department, College of Science, King Saud University, 11451 Riyadh, Kingdom of Saudi Arabia; 3 AP-HP, Department of Rheumatology, Cochin Hospital, Paris, France; 4 Rheumatology Department, Hospital Clinic, IDIBAPS, University of Barcelona, Barcelona, Spain; 5 Department of Social Medicine (Clinical Epidemiology), Federal University of Espirito Santo, Vitória, Brazil; 6 Protein Research Chair, Biochemistry Department, College of Science, King Saud University, 11451 Riyadh, Kingdom of Saudi Arabia; 7 Global Patient Ambassador, Musculoskeletal Research International, Inc., Miami, FL USA; 8 Patient Partner, Holiday, FL USA; 9 EUPATI Fellow, Holiday, FL USA; 10 Clinical Research Experts, LLC., Tampa, FL USA; 11 Research Unit in Public Health, Epidemiology and Health Economics, Department Physical Activity and Rehabilitation Sciences, University of Liège, Liège, Belgium; 12 MRC Lifecourse Epidemiology Centre, University of Southampton, Southampton, UK; 13 Department of Medicine, School of Clinical Sciences, Department of Endocrinology, Monash University, Clayton, VIC Australia; 14 International Osteoporosis Foundation, Geneva, Switzerland; 15 Institute of Musculoskeletal Medicine, LMU University Hospital, LMU Munich, Munich, Germany; 16 Musculoskeletal University Center Munich, LMU University Hospital, LMU Munich, Munich, Germany; 17 Department of Health Services Research, CAPHRI Care and Public Health Research Institute, Maastricht University, Maastricht, the Netherlands; 18 Department of Radiology - Densitometry, Rede Materdei de Saúde, Belo Horizonte, Minas Gerais Brazil; 19 Department of Internal Medicine, Hospital del Mar-IMIM, Autonomous University of Barcelona and CIBERFES, Instituto Carlos III, Barcelona, Spain; 20 Department of Diabetes, Nutrition and Metabolic Disorders, Clinical Pharmacology, University of Liege, CHU de Liège, Liège, Belgium; 21 Protein Research Chair, College of Science, King Saud University, Riyadh, Saudi Arabia; 22 Division of Bone Diseases, Geneva University Hospitals and Faculty of Medicine, Geneva, Switzerland; 23 Department of Trauma, Orthopedics and Reconstructive Surgery, MVZ Endokrinologikum Göttingen, University Medical Center Goettingen, Von-Siebold-Straße 3, Robert-Koch-Straße 40, 37075 Goettingen, Germany; 24 Department of Rheumatology and ULR 44490, University-Hospital of Lille, 59037 Lille Cedex, France; 25 University Vita-Salute San Raffaele, Milan, Italy

**Keywords:** Osteoporosis, Anabolic treatment, Teriparatide, Abaloparatide, Romosozumab, Fragility fracture risk

## Abstract

Bone forming agents, also known as anabolic therapies, are essential in managing osteoporosis, particularly for patients at very high-risk of fractures. Identifying candidates who will benefit the most from these treatments is crucial. For example, this group might include individuals with severe osteoporosis, multiple vertebral fractures, a recent fragility fracture or those unresponsive to antiresorptive treatments. Definitions of patients with a very high fracture risk vary across nations, are often based on fracture history, bone mineral density (BMD), and/or fracture risk calculated by FRAX® or other algorithms. However, for very high-risk patients, anabolic agents such as teriparatide, abaloparatide, or romosozumab are commonly recommended as first-line therapies due to their ability to stimulate new bone formation and improve bone microarchitecture, offering significant benefits in rapid fracture reduction over antiresorptive therapies. The cost-effectiveness of these agents is a critical consideration for decision-makers. Despite their higher costs, their effectiveness in significantly reducing fracture risk and improving quality of life can justify the investment, especially when long-term savings from reduced fracture rates and associated healthcare costs are considered. Additionally, after completing a course of anabolic therapy, transitioning to antiresorptive agents like bisphosphonates or denosumab is crucial to maintain the gains in bone density and minimize subsequent fracture risks. This sequential treatment approach ensures sustained protection and optimal resource utilization. In summary, the effective use of bone forming agents in osteoporosis requires a comprehensive strategy that includes accurate patient identification, consideration of cost-effectiveness, and implementation of appropriate sequential treatments, ultimately maximizing patient outcomes and healthcare efficiency.

## Introduction

Osteoporosis is a bone disease characterized by decreased bone mineral density (BMD) and deterioration of bone microarchitecture, leading to an increased risk of fractures [1, 2]. Often referred to as the “silent disease” because it progresses without obvious symptoms until a fracture occurs [3], osteoporosis poses a significant health challenge, particularly among aging populations worldwide. This condition is particularly prevalent among postmenopausal women, due to oestrogen deficiency, but it can also affect men and younger individuals, especially those with specific medical conditions or lifestyle factors [4]. In Europe, the recent ScoreCard for OsteoPorosis in Europe (SCOPE) collaboration, proposed by the IOF (International Osteoporosis Foundation) has estimated that in the next 10 years, more than five million individuals will be affected by osteoporotic fractures in the European Union plus the UK and Switzerland (EU 27 + 2 countries), a significant increase of about 25% from 2019 [5].

In osteoporosis, increasing attention is given to patients at very high-risk of osteoporotic fractures for whom the most potent treatments and comprehensive monitoring may be appropriate, due to higher potential health outcomes benefits [6]. Unfortunately, only a limited number of these patients are correctly recognized and treated according to the best pharmacological and non-pharmacological treatments, indicating the need to change the paradigm of patients at very high-risk of osteoporotic fractures [7]. Moreover, the situation about access and criteria for prescribing bone forming agents is still convoluted with marked differences country by country. For these reasons, in this paper, we aim to review definitions of very high-risk of fracture, rationale of support, access to bone forming agents across major markets/nations across the world to show the current guidelines and barriers for the optimal use of bone forming agents in patients with very high-risk of fractures.

## Methods

In February 2024, the European Society for Clinical and Economic Aspects of Osteoporosis, Osteoarthritis and Musculoskeletal Diseases (ESCEO) convened a working group to address the issue of the optimal use of bone forming agents in osteoporosis. The working group included clinicians (rheumatologists, endocrinologists, orthopaedic surgeons, geriatricians), epidemiologists, health economists, a patient partner, public health and regulatory experts from several countries across three continents. At the meeting, the latest evidence regarding optimal use of bone forming agents in osteoporosis was reviewed and synthesized with expert opinion to inform a GRADE (Grading of Recommendations Assessment, Development and Evaluation) [8] assessment of statements for the detection of patients at very high-risk of fragility fractures and the use of bone forming agents in osteoporosis. A session was held specifically for patient representation.

Literature searches were performed (by J.Y.R., R.R., O.B., B.C., N.C.H., E.M.C.) and the results were presented to the working group in a series of sessions (rational of the use of bone forming agents, use in representative major markets, economic aspects, side effects) and patient perspectives (from patient representatives). This evidence, together with expert opinion, was used to inform the GRADE assessment.

After reviewing the evidence, the working group undertook a GRADE assessment to determine recommendations for the optimal use of bone forming agents in osteoporosis [9].

The GRADE process involved expert members of the working group (*n* = 21) grading a list of statements (which had been formulated by the core writing group (J.Y.R., R.R., O.B., C.B., N.C.H.) with a level of agreement (‘agree’ or ‘disagree’) and a strength of recommendation (‘recommended’ or ‘not recommended’, rated as ‘strong’ or ‘weak’ depending on the extent to which the member agreed with the statement) based on the considered quality of evidence, magnitude of effect, risk-to-benefit ratio, health economic data, values and preferences. Working group members were allowed to choose the most appropriate category and there was one round of voting. If members did not feel that the statement fell within their area of expertise, it was graded ‘Not qualified’ and if a response was not provided the statement was graded ‘Not recorded’.

## Rationale for the use of bone forming agents in patients at high or very high fracture risk

It is known that osteoporosis and fractures are costly conditions and are recognized as important factors in decreasing intrinsic capacity in older people [10]. Despite the availability of established algorithms for assessment of fracture risk and many proven pharmacological treatments to improve bone mineral density and decrease the risk of fracture, there is still a substantial gap between those individuals warranting assessment and treatment, compared with those actually treated worldwide. For example, it was recently reported that 14.8 million of 21 million European women eligible for intervention are left untreated [5]. Therefore, to increase the rate of coverage of older people, particularly women at high or very high-risk of fractures, treated with medications able to prevent fractures we need to vastly improve efficiencies and robustness of identifying and treating patients [11].

### Overview of anabolic treatments

In this section, we will discuss the literature supporting the use of the bone forming agents, namely teriparatide, abaloparatide, and romosozumab.

From a pharmacokinetic point of view, there appear to be some differences between these three medications. It is known that teriparatide first stimulates bone formation, followed by a later increase in bone resorption; abaloparatide, when compared to teriparatide, shows a lower rate of bone formation and bone resorption, but a higher net bone forming effect [12]. This effect is also reflected by the histomorphometric effects of bone forming agents: abaloparatide and teriparatide increase cortical porosity, whilst romosozumab does not; similarly, abaloparatide seems to have the greatest effect on periosteal surface and teriparatide also has an effect here, to a lesser extent. Romosozumab shows a rapid increase in bone formation with a decrease in bone resorption that is maximum at 3 months, while the anti-resorptive effect remains throughout [13]. Moreover, as shown in another study including 29 bone biopsies, the stimulation of bone formation in the first 2 months of romosozumab treatment in postmenopausal women with osteoporosis is predominately due to increased modeling-based bone formation on endocortical and cancellous surfaces, whilst the action of periosteal surface is limited [13].

Finally, whilst cancellous and cortical bone are increased by teriparatide, they are increased much more with abaloparatide and romosozumab, even if head-to-head comparisons with these three medications are still missing [12].

This initial evidence may affect the physician’s choice about the bone forming agent to use that is also determined by clinical factors such as clinical experience, increase in bone mineral density (BMD), adverse events and costs [14]. Moreover, the clinical characteristics of the studies leading to the approval of bone forming agents may further affect the choice of the most appropriate medication. About teriparatide, for example, the mean age of the participants included was younger than the RCT (randomized controlled trial) leading to the approval of romosozumab [15].

### Efficacy of anabolic treatments

From a clinical perspective, as reported in the VERO trial, at 24 months, new vertebral fractures occurred in 28 (5.4%) of 680 patients in the teriparatide group and 64 (12.0%) of 680 patients in the risedronate group, with a significant decrease of 56% in women treated with teriparatide [16]. However, no definitive data about non-vertebral fragility fractures, such as hip fracture, were available (p = 0.10) [16]. However, in the Fracture Prevention Trial compared with placebo, teriparatide reduced non-vertebral fractures by 53%. For abaloparatide, the ACTIVE (Abaloparatide Comparator Trial In Vertebral Endpoints) RCT shows that among 2463 post-menopausal women abaloparatide was associated with a clinically meaningful reduction in vertebral fracture risk (relative risk, RR = 0.14; 95%CI: 0.05–0.39) compared with placebo [17]. Overall, this effect was maintained after 24 months of alendronate, following abaloparatide [18]. Finally, in a real-world experience derived from US patient claims data from Symphony Health, Integrated Dataverse (IDV)®, over 19 months of follow-up, the risk for hip fractures was reduced by 22% for abaloparatide compared with teriparatide (1.0% vs 1.3%, P = 0.04) [19]. When limited to patients with at least 1 year of consecutive treatment exposure, abaloparatide was associated with a further decrease in the risk of hip fracture [19]. Finally, as demonstrated in a large network meta-analysis, all treatments commonly used for the treatment of osteoporosis, except ibandronate, were associated with beneficial treatment effects relative to placebo, but the use of abaloparatide was associated with the greatest effect on vertebral and non-vertebral fractures compared with the other pharmacological options [20]. In particular, abaloparatide demonstrated the greatest treatment effect relative to placebo in the vertebral fracture network (RR = 0.13; 95% credible interval [CrI] 0.04–0.34), the non-vertebral fracture network (RR = 0.50; 95% CrI 0.28–0.85), and the wrist fracture network (RR = 0.39; CrI 0.15–0.90) [20].

Some data have reported that romosozumab was associated with a lower risk of vertebral fracture than placebo at 12 months and, after the transition to denosumab, at 24 months [21]. Of importance, after one year of treatment, new vertebral fractures had occurred in 16 of 3321 patients (0.5%) in the romosozumab group, as compared with 59 of 3322 (1.8%) in the placebo group (representing a 73% lower risk with romosozumab; P < 0.001). [21] In a post-hoc analysis, romosozumab was associated with a favorable effect in decreasing fracture risk after the transition to denosumab and to a consistent increase in BMD, over 3.5 years of follow-up: most romosozumab-treated patients, in fact, experienced ≥ 3% gains in BMD from baseline at month 12 (spine, 96%; hip, 78%) compared with placebo (spine, 22%; hip, 16%) [22].

In Phase 3 RCTs, compared with placebo, therapy with teriparatide, abaloparatide, or romosozumab resulted in large increases in lumbar spine BMD and smaller increases in hip BMD [23]. The vertebral fracture risk is of clinical importance for all the bone forming agents, whilst the reductions in non-vertebral fracture risk were observed only for teriparatide and abaloparatide compared to placebo [23]. In a large RCT comparing abaloparatide, teriparatide and placebo, abaloparatide seems to be the best option in preventing new vertebral and non-vertebral fractures over 18 months, even if we should take in account that non-vertebral fractures were considered as secondary endpoints and that the study is probably not powered to detect any difference between PTHR1 analogues [17]. Similarly, literature supports that teriparatide and romosozumab lead to a more rapid and greater magnitude increase in BMD compared with bisphosphonates finally leading to larger decreases in fracture risk [16, 24]. In particular, over 2 years of follow-up, a 48% lower risk of new vertebral fractures was observed in the romosozumab-to-alendronate group (6.2% of the initial patients enrolled) than in the alendronate-to-alendronate group (11.9%) (P < 0.001) [24]. In some head-to-head studies, abaloparatide and romosozumab led to a higher increase in BMD at femoral neck and total hip compared with teriparatide [23], but other literature is needed to confirm these findings.

All these findings are of importance in order to better individualize the profile of patients at very high-risk of osteoporotic fractures that require a personalized treatment with bone forming agents [25]. Unfortunately, despite an important literature supporting the efficacy and safety of bone forming agents [26], some barriers are of importance as well as the role of general practitioners should be increased in terms of early recognition of patients at high-risk of osteoporotic fractures and for their follow-up.

Since in daily clinical practice bone forming agents could be used only for a limited period of time, the sequential therapy is of great clinical interest [23]. Indeed, such an approach has been adopted in the trials of abaloparatide and romosozumab, with good evidence for all three anabolics of bone loss following the treatment period, without subsequent antiresorptive therapy. Thus, the clinical expectation is that any course of an anabolic medication will be followed by an appropriate antiresorptive. Given that by definition, these are high fracture risk individuals, it is most likely that this would be a parenteral therapy such as denosumab [27].

## The cardiovascular safety of bone anabolic agents

The cardiovascular profile of bone forming agents is of particular importance for increasing the compliance for these medications and since the prevalence of cardiovascular conditions significantly increases in post-menopausal women in which these medications are largely used.

It has been known for more than 40 years that PTH analogues can cause an increase in heart rate and a decrease in blood pressure and, probably, abaloparatide may induce more palpitations than teriparatide, even if this evidence is limited only to one study [28]. Despite these clinical side effects, the totality of the evidence base, including prospective real-world evidence, strongly suggests that both abaloparatide and teriparatide are safe from a cardiovascular point of view [19]. In this regard, literature supports measurement of blood pressure before starting the treatment with PTH analogues and to sit or lie down at the time of the first injection to mitigate the small risk of orthostatic hypotension [19].

In contrast, the cardiovascular safety of romosozumab has been a key consideration in its implementation [29]. Indeed, in the RCT leading to the approval of romosozumab (ARCH trial), the incidence of new cardiovascular side effects was 2.5% with romosozumab treatment vs. 1.9% in the alendronate arm (p < 0.0001) [24]. The same was observed in BRIDGE study in men where adjudicated serious cardiovascular adverse events occurred in 4.9% men treated with romosozumab and 2.5% placebo treated men [30].

A systematic review with meta-analysis published in 2020 has reported that among patients with primary osteoporosis romosozumab therapy might increase the risk of 4 P (heart failure, myocardial infarction, stroke, death for all causes) MACE (major adverse cardiovascular events) [31]. Although there is some mechanistic evidence possibly linking sclerostin modulation with cardiovascular outcomes, this is far from conclusive in either direction [32]. The EMA (European Medicines Agency) indicated that romosozumab should not be used in women who have previously experienced a myocardial infarction or stroke [33]. After these initial concerns, the cardiovascular safety of romosozumab was further explored by other animal, real-world and intervention studies [34]. As summarized by Turk et al. the totality of the nonclinical data that included a comprehensive toxicology program, additional cardiovascular studies, and data from the literature did not identify a biologically plausible mechanism to explain the increase in cardiovascular side effects driven mainly by myocardial infarction and stroke observed with romosozumab compared with alendronate in the ARCH trial [35]. Post-marketing surveillance data have identified a possible increased risk in romosozumab users in Japan, but wider assessments are pending [36]. Moreover, it has been reported that among those with pre-existing risk factors for cardiovascular outcomes such as those over the age of 80 and patients with co-reported cardiovascular medications, may have a higher risk of fatal CVD events and, therefore, should be used very cautiously in these categories [36]. Probably, as shown in a Pharmacovigilance Analysis of the US Food and Drug Administration Adverse Event Reporting System, romosozumab might lead to a higher risk of MACE, but only among those with pre-existing cardiovascular risk factors for cardiovascular outcomes or taking medications for cardiovascular diseases, and in those aged more than 80 years [36].

In conclusion, there is a substantial evidence base across RCTs and real-world evidence from large datasets, together with preclinical data, confirming the long-term safety of abaloparatide and teriparatide for cardiovascular outcomes. Indeed, the literature supports the notion that cardiovascular assessment at baseline for these therapies should only consist of measurement of blood pressure, or possibly lying and standing blood pressure, rather than any wider evaluation. Overall, we can argue that cardiovascular safety is not a clinically relevant issue for parathyroid hormone receptor agonists and that recent clinical data do not support the need for cardiovascular assessment (except blood pressure measurement) before commencing abaloparatide or teriparatide. Whilst there is a potential cardiovascular risk signal with romosozumab, the mechanistic basis of this has not been confidently established, and further real-world data from post-marketing surveillance studies are awaited. In the meantime, as per the EMA label, it should be avoided in those with a prior myocardial infarction or stroke.

## Identification and treatment of patients at very high-risk of fractures: evidence from representative markets

When we consider Europe (Table 1), it should be noted that huge differences exist in terms of availability and reimbursement of the most common bone forming agents. Abaloparatide, for example, is available only in a few countries in Europe and it should be in the European market between 2024 and 2025 for several other countries. All these data open the important question that some relevant differences exist about the profile of the patients that are assessed and reimbursed and the agencies indicating these criteria. Moreover, as detailed after, relevant differences exist about which criteria and guidelines are used to make the reimbursement: for example, it is estimated that an annual cycle with romosozumab might cost around 7,000 €, whilst teriparatide might cost roughly half as much and for which several (less expensive) biosimilars are available. However, even if international guidelines vary in the definition of very high fracture risk, bone forming agents should be considered as the first option for these patients.

**Table 1:** Definition and management with bone forming agents of patients at very high-risk of fractures across different countries

Country	Criteria for high-risk osteoporotic fractures	Use of FRAX	Medications available	Reimbursement organs
Spain	● > 2 vertebral fractures ● Fractures and low T-score (< −3.0) ● Very low T-score (< −3.5)	Not used	Teriparatide for 24 months; romosozumab for 12 months; abaloparatide for 18 months	Reimbursed by National Health System
France	● Severe/major osteoporotic fractures ● Non-severe osteoporotic fractures with low BMD ● Very high clinical risk of fractures	Yes, particularly in uncertain cases	Teriparatide (for 18 months + other 6 months); abaloparatide and romosozumab still not available	Reimbursed by National Health System and from private insurances after the first 18 months
Germany	● Combination of sex, age, low BMD (T-score) and clinical risk factors > 10% risk to experience an osteoporotic fracture within the next 3 years	Not used	Teriparatide (24 months); romosozumab (12 months); abaloparatide (18 months)	Reimbursed by National Health System
United Kingdom	● FRAX based definition of patients at very high-risk of fractures	Yes	Teriparatide (24 months); romosozumab (12 months); abaloparatide not available	Reimbursed by National Health System
Australia	● T-score <−3.0 ● Recent fracture within 2 years and/or history of 2 or more fragility fractures ● Clinical risk factors (such as use of corticosteroids) ● FRAX result with a risk of a major osteoporotic fracture ≥ 30%, or hip ≥ 4.5%	Yes	Teriparatide (24 months); romosozumab (12 months); Abaloparatide still not available	Reimbursed by National Health System, but the criteria are more restrictive than those used for defining high-risk patients
Brazil	● T-score < −2.5 with vertebral/hip fracture ● Multiple vertebral fractures ● Two or more osteoporotic fractures or with a glucocorticoid induced osteoporosis (GIO) or with T score < −3.0 with another clinical risk factor ● FRAX: 1.2 times above the age-specific thresholds	Yes	Teriparatide (24 months); romosozumab (12 months); Abaloparatide still not available	Private insurances and National Health System (only in cases of therapeutic failure following prior use of antiresorptive agents)

Table 1 shows the most important characteristics of representative markets/nations across the world. Briefly, in Spain patients at very high-risk of fractures are defined as (a) 2 or more vertebral fractures, or equivalent situation (e.g., vertebral and hip fracture) or (b) vertebral or hip fracture together with T-score < −3.0 or (c) very low BMD (T-score < −3.5), according to national guidelines published in 2022 [37]. For women at very high-risk of fractures, in Spain, the actual indication is to give teriparatide or romosozumab. The first option can be guaranteed for 24 months, the second for 12. After this cycle, the patient could receive denosumab or bisphosphonates, based on clinical judgment.

In France, the most recent guidelines are from 2018 [38]. It should be noted that, in this country, therapy is initiated in less than 15% of high-risk patients [39]. This relevant undertreatment is associated with a high economic burden, particularly during the first year [40]. In France, patients at very high-risk of osteoporotic fractures are those reporting severe fracture/major fracture (whatever the BMD level) or non-severe low trauma fracture (depending on BMD level or on risk fracture assessed with the FRAX) or without fractures, but with very low BMD or very high-risk of fracture according to the FRAX. In this context, FRAX is felt of particular importance when non-severe fractures are present with a T-score between −1 and −2 or in the absence of fracture, but a T-score between −2 and −3.

In Germany, very recent guidelines (published at the end of 2023) are available. In this country, the combination of low BMD at the total hip and other relevant risk factors gives a risk stratified according to age and sex in some groups, < 3%, 3–5%, 5–10% and above 10%. In the 3% group, specific osteoporosis drugs can be used, in the 5% group anabolic drugs can be used, in the 10% group anabolic should be used even as first-line. Patients at very high-risk of osteoporotic fractures are defined those with a risk > 10% to suffer an osteoporotic fracture within the next 3 years and these patients are recommended to receive first-line bone forming therapy. On the contrary, patients with a risk between 5 and 10% to experience an osteoporotic fracture within the next 3 years can be considered to be treated with an anabolic drug, e.g. if clinical risk factors are present such as use of corticosteroids, rheumatoid arthritis, or decreased bone microarchitecture ascertained using the trabecular bone score (TBS). Given this approach, integrating BMD, sex, age and risk factors especially the imminent fracture risk, FRAX is not used in Germany.

In the UK, on the contrary, as per previous ESCEO position papers [6, 41], high-risk patients are defined using the FRAX, since this tool is easy to use metric, can be automated (e.g., in general practitioners’ software), linked to 10 year probability of fracture risk, at the heart of many international approaches to risk assessment, and finally, easily incorporated into national guidelines e.g. NOGG (National Osteoporosis Guideline Group) [6]. Moreover, FRAX was recently updated in the FRAXplus version that adds some newer potential risk factors for fractures such as low trabecular bone score BMD or duration of diabetes, and permits modification of FRAX score according to recency and site of prior fracture.

In Australia, there are guidelines published in 2023. The criteria to identify very high-risk patients are several, including: T-score ≤ −3.0 and/or recent fracture within 2 years and/or history of 2 or more fragility fractures.

and/or clinical risk factors (such as use of corticosteroids) and/or FRAX result with a risk of a major osteoporotic fracture ≥ 30%, or hip ≥ 4.5%. However, current reimbursement criteria are restrictive and second-line T-score < −3.0, two fragility fractures, one of which must have occurred after 12 months of antiresorptive therapy, or intolerance to antiresorptive therapy. Newer less restrictive criteria have recently been approved to allow the use of romosozumab as a first-line treatment (BMD T-score of ≤ −2.5, with either: a recent hip or clinical vertebral fracture, or multiple clinical fractures (including one within the last 2 years). Also in this country, an important mismatch between patients eligible for bone forming therapy and those actually treated exists.

Finally, in Brazil, two societies gave important recommendations about osteoporosis in women [42]. Briefly, in this country, very high-risk patients were considered those with a T-score < −2.5 with vertebral/hip fracture; multiple vertebral fractures or two or more osteoporotic fractures; fragility fracture while on long-term glucocorticoid therapy; with T score < −3.0 with another clinical risk factor or finally, with FRAX: 1.2 times above the age-specific thresholds [42]. Similarly, high-risk women were considered those treated with anti-resorptive medications who continue to lose bone and sustain a fragility fracture or sustain two or more fractures during the treatment or those with GIO (glucocorticoid induced osteoporosis) having a fracture despite an optimal treatment.

In summary, this overview demonstrates that, commonly, we have different criteria used for identifying high and very high-risk patients across countries. Inevitably, this means that the average fracture probability of individuals recommended for anabolic therapy will differ between countries. However, there is a commonality in terms of the conceptual approach which prioritises those at highest levels of fracture risk for anabolic therapy. A further consideration is the lack of consideration of men in these approaches, a topic recently addressed by ESCEO [43].

## Use of bone forming agents in patients at high or very high-risk of fractures: barriers and solutions

There are potential barriers to the use of anabolic agents across various considerations including those of the patient experience, cost and logistics. From the patient’s point of view, one of the most common issues is that teriparatide and abaloparatide are administered via a daily subcutaneous administration and romosozumab every month. Whilst the majority of individuals are able to learn to do the injection themselves, it remains a barrier for some. A linked issue is that, for example teriparatide must be kept refrigerated, leading to risks of drug wastage whereas abaloparatide does not need to be refrigerated after the first injection [33].

Clear communication with patients, as ever is key to effective disease management, and messages about the greater magnitude of benefit and speed of action associated with anabolic therapy compared with antiresorptive medications, are important [44]. A pragmatic positive for many patients is the lack of concerns over osteonecrosis of the jaw, that may affect 1 in 1000 patients under treatment with romosozumab, and atypical femoral fractures with anabolic medications. Initial concerns over osteosarcoma as a possible side-effect of PTH analogues, have been convincingly proven to be unfounded (derived from high dose studies in rat models) in clinical use [45]. The Food and Drug Administration only suggests to avoid use in patients with increased risk of osteosarcoma such as those with open epiphyses, metabolic bone diseases including Paget’s disease, bone metastases or history of skeletal malignancies, prior external beam or implant radiation therapy involving the skeleton, and hereditary disorders predisposing to osteosarcoma [46]. The same principles could be applied to abaloparatide.

## The point of view of the patients

Bone forming agents, also known as anabolic therapies, are very important in treating osteoporosis, especially for patients who are at a high-risk of fractures. These patients could be those with severe osteoporosis, multiple spinal fractures, a recent fracture due to a minor fall, or those who haven’t responded to other treatments.

The definition of “high fracture risk” can vary across different countries and is often based on factors like previous fracture history, bone density, and calculated fracture risk using tools like FRAX®.

For these high-risk patients, anabolic agents like teriparatide, abaloparatide, or romosozumab are often recommended as the first choice of treatment. These drugs can stimulate new bone formation and improve the structure of the bone, providing significant benefits in quickly reducing the risk of fractures compared to other treatments.

While these drugs are more expensive, their effectiveness in significantly reducing fracture risk and improving quality of life makes them a worthwhile investment. This is especially true when considering the long-term savings from reduced fracture rates and associated healthcare costs.

After completing a course of anabolic therapy, patients continue therapy by switching to other drugs like bisphosphonates or denosumab to maintain the increase in bone density and minimize the risk of future fractures. This approach of using different treatments in sequence ensures ongoing protection and makes the best use of resources.

In summary, the effective use of bone forming agents in osteoporosis involves a comprehensive strategy that includes accurately identifying the right patients, considering cost-effectiveness, implementing the right sequence of treatments whilst actively involving patients and ensuring they understand how to best manage the expected and potential side effects. This approach aims to maximize patient outcomes and healthcare efficiency.

## Health economic data supporting sequential treatment with bone forming agents in patients at high or very high fracture risk

The economic aspects are of importance to support the use of bone forming agents in patients affected by osteoporosis, particularly from a public health perspective. A systematic review published in 2023 shows that sequential treatment was cost-effective and sometimes even dominant compared to monotherapy [27]. In particular, this work shows that the sequential therapy with teriparatide after alendronate use was not cost-effective compared to alendronate, probably because these studies were made among patients not at high-risk of fractures [27]. On the contrary, the use of sequential treatment with romosozumab or abaloparatide followed by alendronate in monotherapy was cost-effective compared to alendronate [47]. The use of sequential treatment with abaloparatide was dominant compared to sequential treatment with teriparatide [27, 47].

Literature supports that three determinants are of importance for the cost-effectiveness of bone forming agents, i.e., drug cost, age and the severity of osteoporosis. Briefly, literature supports the idea that cost-effectiveness is improved after the age of 60 years compared with younger patients as well as in patients at very high-risk of fractures with a recent fracture and a densitometric osteoporosis [27]. Finally, more recent literature is supporting the idea that the sequential treatment with bone forming agent is cost effective compared to alendronate monotherapy also in men, even if this new evidence is mainly limited to the USA [48].

## Summary of recommendations and guidelines

Here we summarize the recommendations of the working group for the optimal use of bone forming agents in patients at high and very high-risk of fractures. The statements supported by the working group are itemized below and detailed ratings are presented in Table 2. Each statement was presented with the inquiry: “Do you agree with the statement?” accompanied by four possible responses: “Strong do”, “Weak do”, “Weak don’t”, and “Strong don’t”. Consensus on each statement, either in support or opposition, required at least 75% of the Voting Panel to be in either the “do” or “don’t” categories, regardless of strength. If this threshold was not met, no consensus was declared, and no statement was issued. A statement was classified as “strong” if at least 75% of the Voting Panel members rated it as “strong do”. Of course, panel members could abstain from voting due to a conflict of interest or lack of sufficient expertise to evaluate the statement.

- Bone Forming Agents lead to a more rapid, and greater magnitude, increase in BMD compared with oral anti-resorptive medications.
- Bone Forming Agents lead to a more rapid, and greater magnitude, decrease in fracture risk, compared with oral anti-resorptive medications.
- There are no currently available data allowing direct comparison of the anti-fracture efficacy of parathyroid hormone receptor agonists with romosozumab.
- The reduction in risk of vertebral fractures appears similar for abaloparatide and teriparatide.
- Based on the Active, ActivExtend studies and real-world evidence data obtained from US health claims, abaloparatide may have a greater anti-fracture efficacy, compared with teriparatide, for non-vertebral fractures.
- Commencing treatment with a bone forming agent, followed by anti-resorptive maintenance, is likely to prevent more fractures than initial anti-resorptive treatment.
- Whilst international guidelines vary in their definition of very high fracture risk, bone forming agents should be considered as a first-line therapy in such patients.
- ESCEO-IOF position supports the use of absolute fracture probability (FRAX®) and age-dependent intervention thresholds.
- Cardiovascular safety is not a clinically relevant issue for parathyroid hormone receptor agonists (abaloparatide and teriparatide).
- Recent clinical data do not support the need for cardiovascular assessment (except blood pressure measurement) before commencing abaloparatide or teriparatide.
- Patients receiving abaloparatide or teriparatide should be able to sit or lie down at the time of the first injection to mitigate the small risk of orthostatic hypotension.
- Romosozumab is contraindicated in the presence of a prior myocardial infarction or stroke and should be used with caution in older patients with prevalent cardiovascular risk factors.
- Health economic studies support the cost-effectiveness of anabolic first approaches in patients at very high-risk of fractures (independently of the strategy used for their identification).

**Table 2:** Recommendations for an optimal use of bone forming agents in osteoporosis

Recommendation	Strong do	Weak do	No rec	Weak don’t	Strong don’t
Bone Forming Agents lead to a more rapid, and greater magnitude, increase in BMD compared with oral anti-resorptive medications	18	0	0	0	0
Bone Forming Agents lead to a more rapid, and greater magnitude, decrease in fracture risk, compared with oral anti-resorptive medications	18	0	0	0	0
There are no currently available data allowing direct comparison of the anti-fracture efficacy of parathyroid hormone receptor agonists with romosozumab	14	3	1	0	0
The reduction in risk of vertebral fractures appears similar for abaloparatide and teriparatide	16	1	1	0	0
Based on the Active, ActivExtend studies and real-world evidence data obtained from US health claims, abaloparatide may have a greater anti-fracture efficacy, compared to teriparatide, at non-	14	2	1	1	0
Commencing treatment with a Bone Forming Agent, followed by anti-resorptive maintenance, is likely to prevent more fractures than initial anti-resorptive treatment	18	0	0	0	0
Whilst international guidelines vary in their definition of very high fracture risk, Bone Forming Agents should be considered as a first-line therapy in such patients	18	0	0	0	0
ESCEO-IOF position supports the use of absolute fracture probability (FRAX¬∞) and age- dependent intervention thresholds	15	1	1	1	0
Cardiovascular safety is not a clinically relevant issue for parathyroid hormone receptor agonists (abaloparatide and teriparatide)	15	2	0	1	0
Recent clinical data do not support the need for cardiovascular assessment (except blood pressure measurement) before commencing abaloparatide or teriparatide	16	2	0	0	0
Patients receiving abaloparatide or teriparatide should be able to sit or lie down at the time of the first injection to mitigate the small risk of orthostatic hypotension	15	3	0	0	0
Romosozumab is contraindicated in the presence of a prior myocardial infarction or stroke and should be used with caution in older patients with prevalent cardiovascular risk factors	17	1	0	0	0
Health economic studies support the cost-effectiveness of anabolic first approaches in patients at very high risk of fractures (independently of the strategy used for their identification)	13	4	1	0	0

All the sentences were strong recommendations (i.e., 75% of voters selected ‘strong do’), except the last sentence about health economic studies (weak recommendation).

## Conclusions

Bone forming agents, or anabolic therapies, play a crucial role in the treatment of patients with severe osteoporosis, particularly for patients at high-risk of fractures. These recommendations are vital for healthcare providers, policymakers, and researchers aiming to optimize treatment outcomes and resource allocation. The first step is to accurately identify patients who will benefit most from anabolic therapies. The definitional approach varies between countries, based on fracture history, BMD scores, and the use of validated tools to predict fracture risk, such as FRAX.

For patients at very high-risk of fractures, anabolic agents (teriparatide, abaloparatide, romosozumab) are recommended as first-line therapies. The cost-effectiveness of bone forming agents is a critical consideration, also in the light of reimbursement criteria. While these therapies are often more expensive than traditional antiresorptive treatments, their ability to substantially reduce fracture risk and improve quality of life justifies the investment. Economic evaluations should consider the long-term savings from reduced fracture rates, associated healthcare costs and lived patient experience dossiers detailing the magnitude of meaningful effect on endpoints that matters most for patients. Finally, it is mandatory to clearly inform and to actively involve patients when initiating these therapies to ensure their understanding of how to best manage expected and/or potential side effects.

Finally, the effective use of bone forming agents in osteoporosis requires a comprehensive approach that includes identifying high-risk patients, considering cost-effectiveness, and implementing appropriate sequential treatments. These strategies are crucial for maximizing patient outcomes and ensuring the efficient use of healthcare resources across different healthcare systems.

## Data Availability

No datasets were generated or analysed during the current study.
